# Lenvatinib‐Associated Non‐Tuberculous Mycobacterial Pulmonary Disease: A Case Report

**DOI:** 10.1002/rcr2.70316

**Published:** 2025-08-20

**Authors:** Qian Wang, Lili Du, Yiming Wang, Longhui Zhang

**Affiliations:** ^1^ Department of Respiratory and Critical Care Medicine Huangshan City People's Hospital Huangshan Anhui China

**Keywords:** adverse drug reaction, Lenvatinib, nontuberculous mycobacteria

## Abstract

A 64‐year‐old man, with a history of hepatocellular carcinoma and long‐term oral lenvatinib treatment, was hospitalised in our institution twice due to ‘cough’. During hospitalisation, the patient underwent multiple bronchoscopies, with lavage fluid sent for pathogen testing, which returned negative results. Finally, *Mycobacterium kansasii* was detected via next‐generation sequencing (NGS). Considering the patient's poor hepatic reserve, we decided against initiating targeted antimicrobial therapy and discontinued lenvatinib. Two months after stopping lenvatinib, the patient's symptoms of cough and chest tightness improved. A follow‐up chest computed tomography (CT) on March 18, 2025, showed resolution of the lesions.

## Graphical abstract

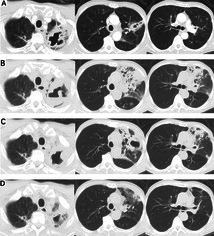

## 1 Introduction

Lenvatinib is an oral protein receptor tyrosine kinase inhibitor approved as a first‐line treatment for unresectable hepatocellular carcinoma [1]. While it provides clinical benefits, lenvatinib inevitably induces adverse events, including hypertension, diarrhoea, myalgia, abdominal pain, proteinuria and so on [2]. To date, there have been no reported cases of lenvatinib‐associated non‐tuberculous mycobacterial (NTM) pulmonary disease. This article presents a case of a hepatocellular carcinoma patient who developed pulmonary NTM infection after long‐term lenvatinib therapy, as detected by next‐generation sequencing (NGS). Symptoms improved and follow‐up CT showed resolution after discontinuation of lenvatinib for 2 months. The case is reported below with a literature review.

## 2 Case Report

A 64‐year‐old male with a 2‐year history of hepatocellular carcinoma (underwent liver resection in March 2022, experienced intrahepatic recurrence 6 months postoperatively, and received transarterial chemoembolization in October 2022, followed by continuous lenvatinib therapy) was hospitalised twice for pulmonary disease.

**FIGURE 1:**
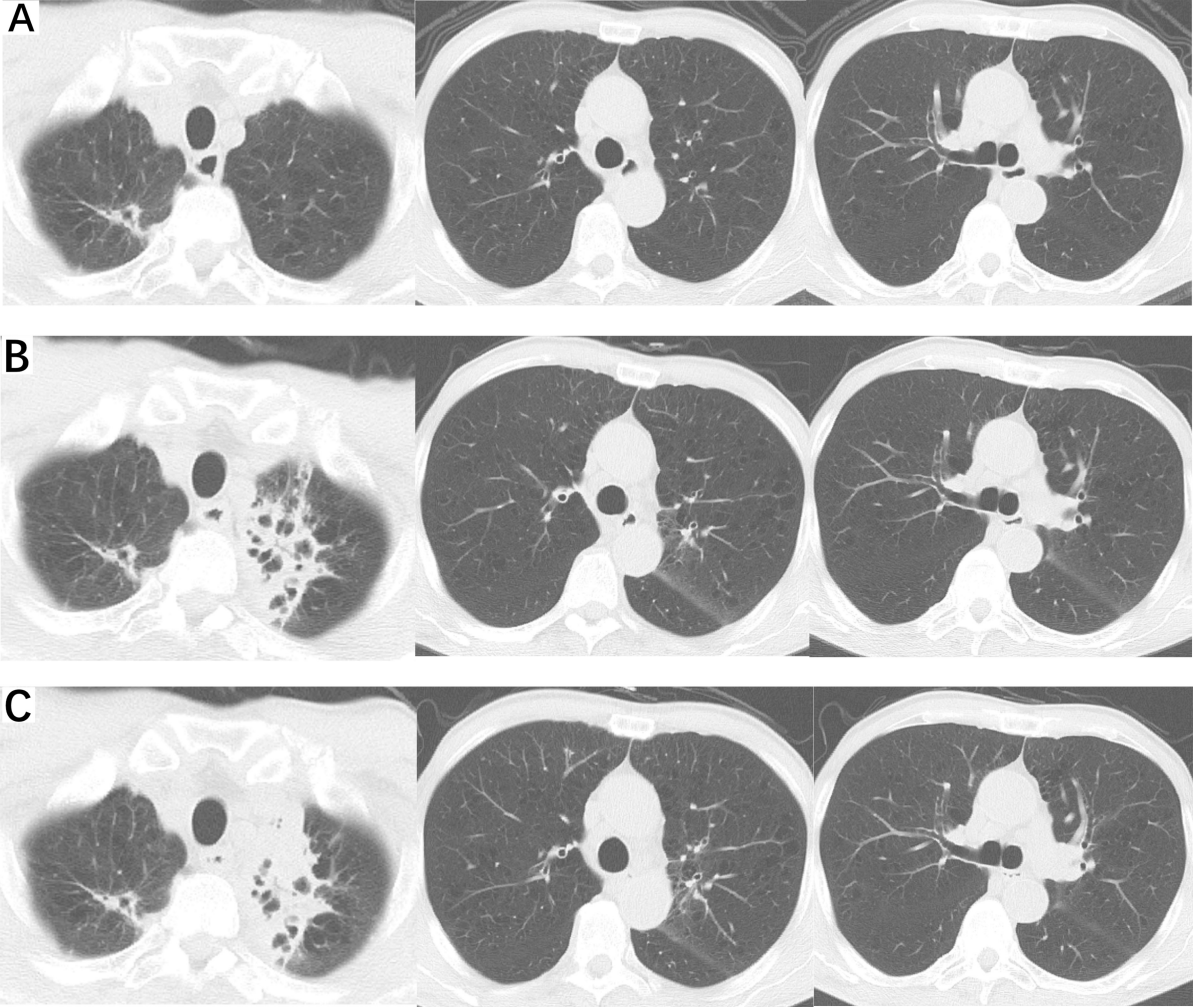
Chest computed tomography (CT) on March 12, 2024 showed roughly normal lungs (A). Chest CT on June 12, 2024 revealed patchy opacities with cavitation in the left lung (B). Chest CT on June 21, 2024 showed the lesion was not absorbed after 7 days of antibiotic therapy (cefoperazone‐sulbactam plus doxycycline) (C).

On June 14, 2024, he was admitted for ‘persistent cough for over one month’. The patient reported dry cough without fever, night sweats or dyspnea but with left‐sided chest pain. No prior treatment was sought. Outpatient chest CT (June 12, 2024) revealed patchy opacities with cavitation in the left lung (Figure 1B), and the patient required hospitalisation due to the progression of the previous CT scan (Figure 1A). On the first day of admission (June 15, 2024), blood tests showed that C‐reactive protein (CRP) was elevated (51.06 mg/L), erythrocyte sedimentation rate (ESR) was elevated (40 mm/h), tuberculosis (TB) IgG antibody was negative, and mycoplasma IgM antibody was weakly positive, and cefoperazone‐sulbactam was treated with doxycycline. On the third day of admission (June 17, 2024), Bronchoscopy showed patent airways without masses, and bronchoalveolar lavage (BAL) from the left upper lobe was sent for microbiological and cytological analysis, and the results were negative. Test Mllineutrophil cytoplasmic antibodies was negative. After Alpha‐Fetal Protein (AFP) and abdominal CT examination, HCC was considered to be stable, and lung lesions were not considered to metastasize. After 7 days of antibiotic therapy, repeat CT (June 21, 2024) showed no improvement (Figure 1C). The patient declined further bronchoscopy and was discharged with symptom relief.

**FIGURE 2:**
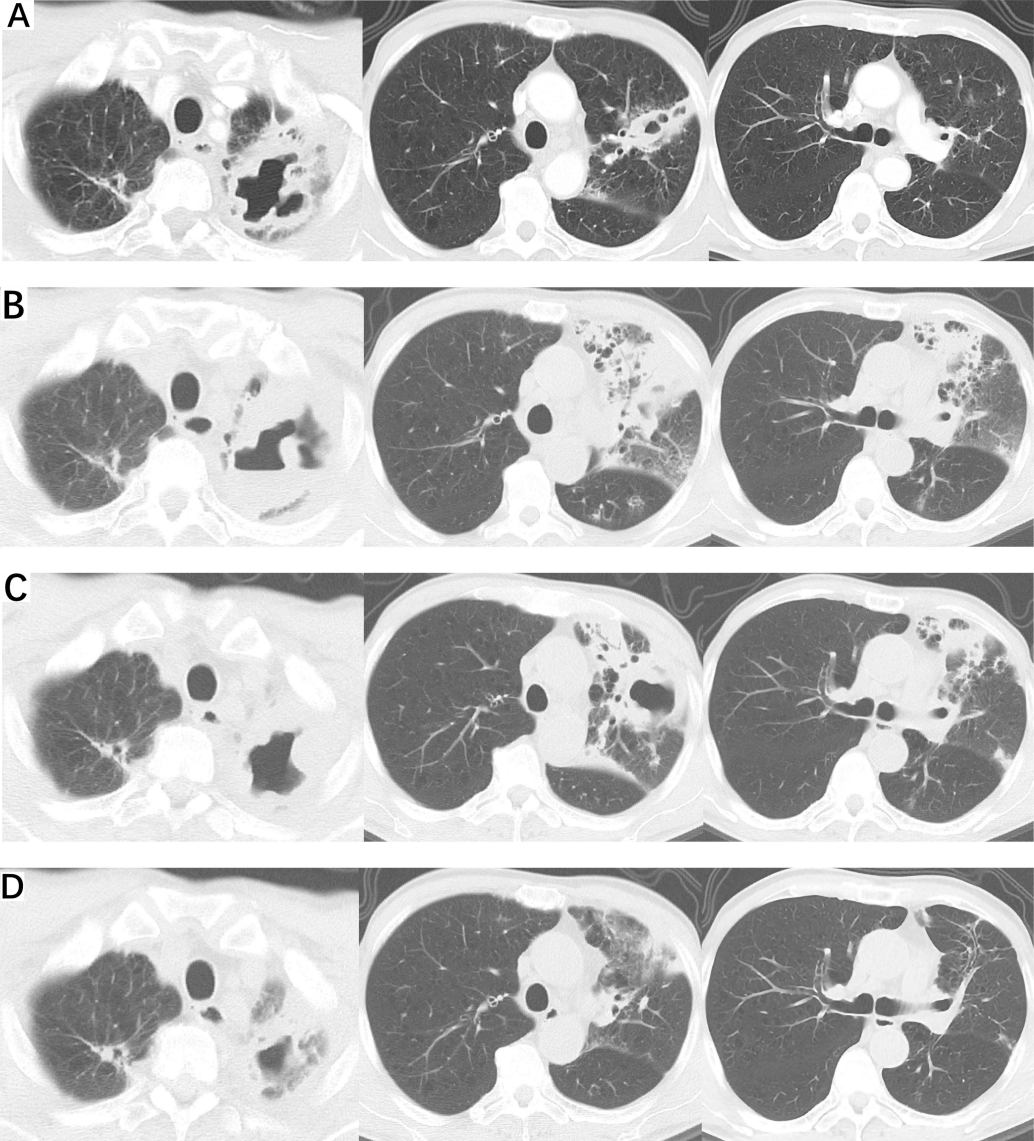
Chest CT on December 18, 2024, demonstrated progression of the left upper lobe cavitary lesion after 6 months of untreated lungs (A). Chest CT on January 3, 2025, showed lesion progression after antibiotic therapy (Levofloxacin first, then linezolid plus cefoperazone sulbactam) (B). Chest CT on January 9, 2025, showed the lesion was not absorbed after antibiotic therapy (linezolid plus cefoperazone sulbactam) (C). Chest follow‐up CT on March 18, 2025, showed significant resolution of pulmonary lesions after two months stopping lenvatinib (D).

On December 18, 2024, the patient was readmitted to the hospital. The patient returned for recurrent cough, productive sputum, fever (up to 39°C), and exertional dyspnea. Chest CT demonstrated progression of the left upper lobe cavitary lesion (Figure 2A). Laboratory data showed CRP was elevated (188.62 mg/L), ESR was elevated (52 mm/h) and AFP was normal. But that for TB IgG antibody returned positive results. Treatment with levofloxacin was started. Repeat bronchoscopy (December 24, 2024) revealed purulent secretions and hypertrophic mucosa in the left upper lobe. Tests for GeneXpert MTB/RIF and tuberculosis of BAL returned negative results. The tracheoscopic pathological result was chronic bronchial inflammation with squamous metaplasia. The NGS of metagenomes (mNGS) of BAL identified *Streptococcus parasanguinis* with 148 sequences and *Mycobacterium kansasii* with 10 sequences detected. Antibiotics were adjusted to linezolid with cefoperazone‐sulbactam on December 28, 2024. Despite treatment, follow‐up CT (January 3, 2025) showed lesion progression (Figure 2B). Considering the patient's body temperature was normal, the treatment was not changed. The CT re‐examination (January 9, 2025) indicates the lesion was not absorbed (Figure 2C). We have discontinued all antibiotics and plan to perform a bronchoscopy again. A third bronchoscopy (January 10, 2025) with targeted capture of NGS (tNGS) confirmed *Mycobacterium kansasii* with 2984 sequences detected. Finally, we diagnosed it as Pulmonary NTM ( *Mycobacterium kansasii* ) disease.

It is worth noting that the patient underwent three bronchoscopic examinations, with BAL sent for TB culture each time. TB culture typically takes 2–3 months. August 20, 2024 BAL fluid culture result (sample collected on June 17, 2024): No *Mycobacterium tuberculosis* growth. February 20, 2025 BAL fluid culture result (sample collected on December 24th, 2024): Nontuberculous mycobacteria detected. March 31, 2025 BAL fluid culture result (sample collected on January 10, 2025): Nontuberculous mycobacteria detected. The results of the last two tuberculin culture tests indicated NTM infection, further confirming our diagnosis.

Given the patient's compromised liver function, anti‐NTM therapy was deferred. Lenvatinib was discontinued. Follow‐up (March 18, 2025) CT showed significant resolution of pulmonary lesions (Figure 2D).

## 3 Discussion

Lenvatinib is primarily indicated for the treatment of unresectable HCC. The drug‐related adverse events predominantly involve the digestive, nervous, urinary systems, as well as skin and subcutaneous tissues, with common adverse reactions including hypertension, diarrhoea, myalgia, abdominal pain, proteinuria and so on [1, 2]. To date, there have been no reported cases of lenvatinib‐associated pulmonary NTM infection. This case presents an HCC patient on long‐term lenvatinib therapy who developed slowly progressive pulmonary lesions diagnosed as *Mycobacterium kansasii* infection. Following discontinuation of lenvatinib without additional antimicrobial therapy, the pulmonary lesions gradually improved, suggesting a potential association between lenvatinib and pulmonary NTM infection.

Lenvatinib exerts its therapeutic effects by targeting vascular endothelial growth factor receptors 1–3, fibroblast growth factor receptors 1–4, and platelet‐derived growth factor receptor‐alpha, and enhances anti‐tumour immune responses through remodelling the tumour immunosuppressive microenvironment [3]. This mechanism may potentially impair immune cell function and activity, leading to compromised cellular immunity. NTM are opportunistic pathogens that can invade and cause infection when host immunity is impaired, either from latent states or environmental exposure [4]. The precise mechanism by which lenvatinib affects immune function remains unclear and warrants further investigation.

NGS technologies, including mNGS and tNGS, have been increasingly applied in clinical practice for infectious diseases [5]. This method can identify species‐level differentiation and offers the highest resolution for bacterial species detection. With the increasing popularity and decreasing costs of NGS technology, it is playing an increasingly significant role in NTM diagnosis [6, 7]. Compared with mNGS, tNGS offers greater economic value by simultaneously detecting both DNA and RNA in a single assay with lower sequencing data requirements, thereby reducing costs [8]. Studies have demonstrated that tNGS yields significantly higher reads per million values for *Mycobacterium tuberculosis* detection compared to mNGS [9], which is consistent with our case where tNGS reported higher sequence counts for *Mycobacterium kansasii* than mNGS. In this case, the patient was identified with the same species of NTM through two NGS tests. Combined with the patient's clinical manifestations (cough, sputum production and fever), chest imaging findings (patchy lung opacities, some with cavitation), laboratory findings (two mycobacterial culture results indicating NTM infection) and treatment course (comparing the outcomes of different interventions at two stages—progression of pulmonary lesions during broad‐spectrum antibiotic use from December 19, 2024, to January 9, 2025, versus improvement after discontinuation of lenvatinib from January 10 to March 18, 2025), the observed clinical improvement after stopping lenvatinib supports the final diagnosis of pulmonary NTM disease.

NTM refers to a large group of mycobacterial infections excluding the *Mycobacterium tuberculosis* complex and *Mycobacterium leprae* , with pulmonary NTM disease being the most common clinical manifestation [10]. The predominant pathogenic species causing pulmonary NTM disease include *Mycobacterium avium* complex, *Mycobacterium abscessus* and *Mycobacterium kansasii* [11]. In this case, the patient was infected with *Mycobacterium kansasii* . According to the ‘Treatment of Nontuberculous Mycobacterial Pulmonary Disease: An Official ATS/ERS/ESCMID/IDSA Clinical Practice Guideline [4]’, the recommended treatment regimen consists of isoniazid or a macrolide combined with rifampin and ethambutol. However, due to the patient's poor hepatic reserve, we weighed the risks and benefits and decided against initiating anti‐NTM drug therapy. Instead, lenvatinib was discontinued. A follow‐up chest CT on March 18, 2025, showed resolution of the pulmonary lesions.

In conclusion, healthcare professionals should maintain heightened clinical vigilance for pulmonary NTM infection in patients receiving lenvatinib therapy. When NTM infection is suspected, tNGS testing is recommended as a cost‐ and time‐efficient diagnostic approach. Tailored therapeutic strategies should be implemented to ensure optimal patient outcomes and safety.

## Author Contributions

Qian Wang and Lili Du prepared the manuscript, which was reviewed by all co‐authors. All authors have approved the final version of the manuscript for submission.

## Consent

The authors declare that written informed consent was obtained for the publication of this manuscript and accompanying images using the consent form provided by the Journal.

## Conflicts of Interest

The authors declare no conflicts of interest.

## Data Availability

The data that support the findings of this study are available on request from the corresponding author. The data are not publicly available due to privacy or ethical restrictions.
